# Assessment of Antibodies Induced by Multivalent Transmission-Blocking Malaria Vaccines

**DOI:** 10.3389/fimmu.2017.01998

**Published:** 2018-01-19

**Authors:** Vinay Menon, Melissa C. Kapulu, Iona Taylor, Kerry Jewell, Yuanyuan Li, Fergal Hill, Carole A. Long, Kazutoyo Miura, Sumi Biswas

**Affiliations:** ^1^Jenner Institute, University of Oxford, Oxford, United Kingdom; ^2^IMAXIO, Lyon, France; ^3^Laboratory of Malaria and Vector Research, National Institute of Allergy and Infectious Disease, National Institutes of Health, Rockville, MD, United States

**Keywords:** malaria, vaccines, dual-antigen, transmission-blocking, viral-vectors, antibodies

## Abstract

A malaria transmission-blocking vaccine would be a critical tool in achieving malaria elimination and eradication. By using chimpanzee adenovirus serotype 63 and modified vaccinia virus Ankara viral vectored vaccines, we investigated whether incorporating two antigens into one vaccine would result in higher transmission-reducing activity than one antigen. We demonstrated that when Pfs25 was administered with other antigens Pfs28 or Pfs230C, either concurrently as a mixed vaccine or co-expressed as a dual-antigen vaccine, the antibody response in mice to each antigen was comparable to a monoantigen vaccine, without immunological interference. However, we found that the transmission-reducing activity (functional activity) of dual-antigen vaccines was not additive. Dual-antigen vaccines generally only elicited similar transmission-reducing activity to monoantigen vaccines and in one instance had lower transmission-reducing activity. We found that despite the lack of immunological interference of dual-antigen vaccines, they are still not as effective at blocking malaria transmission as Pfs25-IMX313, the current leading candidate for viral vectored vaccines. Pfs25-IMX313 elicited similar quality antibodies to dual-antigen vaccines, but higher antibody titers.

## Introduction

Malaria is a parasitic disease with devastating global health consequences. Malaria incidence is estimated at 214 million cases per year, and mortality has been estimated at 438,000 deaths per year (1). There is a critical need for effective malaria vaccines, especially with new global ambitions for malaria elimination and eradication (2), and the decreasing efficacy of existing malaria control interventions due to drug and insecticide resistance (3, 4). The most clinically advanced malaria vaccine candidate, RTS,S, has completed a phase III clinical trial, demonstrating relatively short-lived protection of 46% against clinical malaria and 34% against severe malaria in children and older infants (5). A recent 4-year follow-up study, which included a booster dose, showed a further reduction in efficacy over time (6). Thus, more effective second-generation vaccines are urgently needed especially those which reduce transmission and incidence, rather than simply reducing morbidity and mortality (7). Transmission-blocking vaccines (TBVs) are widely considered an essential tool for malaria elimination, either on their own or as components of a multistage vaccine or other control interventions (8, 9). TBVs elicit antibodies that target sexual-stage antigens of the *Plasmodium* parasite or mosquito antigens when taken up by the mosquito, thereby blocking parasite development and preventing the vector from transmitting the disease. Pfs25, the leading TBV candidate antigen, is a 25-kDa protein expressed on the surface of zygotes and ookinetes in the mosquito midgut (10). Pfs25 has elicited high antibody titers and transmission-blocking activity (TBA) in preclinical animal studies (11). In humans, exceptionally high antibody titers against Pfs25 have been required to achieve effective transmission-reducing activity (TRA; reduction in oocyst density) (12). This has been a major hurdle to further clinical development of TBVs.

Various methods have been utilized to express Pfs25 in an immunogenic form, with variable results, including protein-in-adjuvant formulations, protein conjugate vaccines, DNA vaccines, virus-like particles, and recombinant viral vectors (13). In a phase I clinical trial in 2008, a Pfs25 protein formulated in Montanide ISA51 adjuvant vaccine demonstrated significant TRA, but this required very high antibody titers. Unfortunately, the trial was halted due to safety concerns related to the specific antigen–adjuvant combination (14). We have recently shown that fusion of Pfs25 with a novel molecule IMX313, derived from the oligomerization domain from chicken complement inhibitor protein C4b-binding protein (15), expressed from either viral vectors or as protein–nanoparticles, had significantly higher immunogenicity and increased TRA compared to monomeric Pfs25 (16). Another potential candidate, Pfs230C has recently demonstrated TBA (reduction in prevalence of infected mosquitoes) comparable to Pfs25 (17). Pfs230C is a portion of the antigen Pfs230, which is expressed in the gamete and gametocyte stages of *Plasmodium falciparum* (17, 18). In previous studies, we have shown that Pfs25 and Pfs230C antigens induce the most efficacious antibodies expressed in viral vectors (16, 17).

Another promising approach to TBV development could be to incorporate multiple antigens into one vaccine. Antibodies against Pfs28 alone have shown TBA/TRA (19, 20), and previous studies have shown potential synergy between Pfs25 and Pfs28 (19). Therefore, to inform antigen selection for clinical development advancement, it is important to determine whether antigen combination would be able to increase and/or enhance efficacy as opposed to the use of a single antigen. While several studies with virus and bacterial vaccines have shown interference, to the best of our knowledge, there are limited published studies investigating the combination of antigens in malaria. Same stage antigen combinations that have been tested for blood stage, MSP1 and AMA1, have shown evidence of immune interference by the most immunodominant antigen (21). In addition, Forbes et al. (22) have shown that mixing viral vectors expressing another combination of malaria antigens, CSP and MSP, had no impact on antibody responses to either antigen but immune interference was observed with cell-mediated immunity. However, a study mixing CSP and AMA1 in a DNA-adenovirus prime-boost regimen showed sterile protection mediated by cellular immunity with no interference reported (23). Furthermore, other studies in influenza have shown that co-administration of different antigens improves and induces a broad range of responses (24, 25). The only published transmission-blocking antigen combination studies involving Pfs25 and Pfs28 showed no evidence of positive interference albeit negative interference (19, 26). Thus, we hypothesized that interference, positive or negative, might be antigen dependent and hence needed to test whether combining Pfs25 and Pfs230C for instance would result in immune interference. Thus, to replicate the Ps25 and Pfs28 synergy studies (19, 26, 27), we tested in addition to Pfs230C the possibility of dual-antigen combinations for Pfs25.

Here, we sought to investigate whether antigen combinations would result in increased efficacy. Thus, we investigated the immunogenicity and TRA of dual-antigen TBVs. We used the clinically relevant recombinant viral vectors, chimpanzee adenovirus 63 (ChAd63) and modified Vaccinia Ankara (MVA), in a heterologous prime-boost regime. In multiple preclinical studies, these viral vectors have consistently induced antibodies against TBV candidate antigens that exhibited TRA in standard membrane-feeding assays (SMFAs) (17, 18). Recombinant viral vectors induce functional antibodies in animal studies (18) and are safe and well tolerated in humans (28, 29). We investigated whether there is benefit to include multiple antigens in a TBV using different methods: co-administration of viral vectors expressing two antigens, mixing of vectors prior to administration, and co-expression of two antigens from the same recombinant vector. Antigens were expressed as dual-antigen viral vectored vaccines using either a glycine–proline (GP) linker or a 2A sequence. With a GP linker, the two antigens are expressed as a single fusion protein with a flexible peptide linker between them, and both antigens have been shown to be immunogenic (30). A 2A linker, a 19 αα proteinase encoded by foot and mouth virus, which self-cleaves at the *C*-terminus between glycine and proline residues, was used to express the polyprotein antigen so that each constituent antigen is generated as a separate product (31).

Here, we first report the immunogenicity in mice after either co-administration of two antigens or mixing of antigens before delivery. We also report TRA in SMFAs after mixing immunized serum against two different antigens. We then report immunogenicity and functionality of antibodies (TRA in SMFAs), induced by vectored vaccines designed to co-express two antigens (Pfs25 with either Pfs28 or Pfs230C, with either a GP or 2A linker), compared to monoantigen vaccines. We also report how dual-antigen vaccines compare to Pfs25-IMX313 vaccine, the leading antigen for viral vectored vaccines to date, in terms of immunogenicity and TRA (16).

## Materials and Methods

### Design and Generation of Recombinant Viral Vectored Vaccines

Antigen sequences for Pfs25 (GenBank accession no: AAN35500, αα 22–192), Pfs28 (GenBank accession no: L25843.1, αα 24–196), and Pfs230C (GenBank accession no: PF3D7_0209000, αα 443–1132) were obtained from the NCBI protein database. The predicted *N*-glycosylation sites were changed from Asn-Xaa-Ser/Thr to Gln-Xaa-Ser/Thr as previously described (17, 18). The antigen sequences were codon optimized for expression in humans (GeneArt^®^ Thermo Fisher Scientific, Germany). The predicted native signal peptide was replaced with human tissue plasminogen activator signal peptide sequence (GenBank accession no. K03021) as previously described (32).

For the dual-antigen vaccine constructs, codon-optimized DNA plasmids containing the dual-antigen constructs (Pfs25-GP-Pfs28, Pfs25-GP-Pfs230C, and Pfs25-2A-Pfs230C) were obtained from ThermoFisher Scientific. The Pfs25, Pfs28, Pfs230C, Pfs25-GP-Pfs28, Pfs25-GP-Pfs230C, Pfs25-2A-Pfs230C, and Pfs25-IMX313 inserts were subcloned into the respective ChAd63 and MVA destination and shuttle vectors and recombinant viral vectored vaccines were generated as previously described (17, 18, 33).

### Animal Studies and Vaccinations

All animal experiments, procedures, and handling were performed according to the UK Animals (Scientific Procedures) Act Project License (PPL 30/2414 and 30/2889) and approved by the Oxford University Local Ethical Review Committee. Age-matched female BALB/c mice (Harlan, UK), housed in specific-pathogen free environments, were vaccinated *via* the intramuscular (i.m.) route using a heterologous prime-boost viral vector regime. In all experiments (Table S1 in Supplementary Material), mice were vaccinated at day 0 with a ChAd63 priming dose of 1 × 10^8^ IFU and boosted at day 56 with a MVA dose of 1 × 10^7^ PFU expressing the recombinant antigens.

When two different antigens were co-administered (Co-ad) in this study, 1 × 10^8^ IFU of ChAd63 of each antigen was delivered i.m. in different limbs of the animal (and the same for the MVA boost). When two different antigens were mixed in this study, 1 × 10^8^ IFU of ChAd63 and 1 × 10^7^ PFU of MVA of each antigen were premixed in a syringe and then delivered as a single vaccine.

Control immunizations were performed with ChAd63 and MVA expressing green fluorescent protein (GFP). Vaccines were prepared in sterile, endotoxin-free PBS (Invitrogen, UK). Antibody responses to the vaccine antigens were assessed at days 14, 55, and 70.

### Western Blot Analysis

To determine the expression of the recombinant antigens expressed by the viral vectored vaccines in mammalian cells, 1 × 10^7^ cells/ml of HEK293 cells were seeded onto six-well plates and transfected with pENTR4-LPTOS shuttle plasmid DNA (expressing each of the recombinant antigens detailed above), using Lipofectamine™ 2000 (Invitrogen, UK). Cells were incubated for 48 h at 37°C and 5% CO_2_. Supernatants and cell lysates were harvested for Western blot analysis, by standard methods (34). In brief, polyacrylamide gel electrophoresis was performed, samples were transferred to blotting membrane, blots were incubated with the respective primary antibodies, and blots were then washed with PBS/T. After washing, the blots were incubated with alkaline phosphatase-conjugated donkey anti-mouse IgG secondary antibodies (Jackson Immuno Research, USA) and washed in PBS/T. Blots were rinsed briefly in deionized water, and then the protein bands were stained and detected using BICP^®^/NBT alkaline phosphatase substrate (Sigma-Aldrich, UK). Prestained protein ladders (NEB UK) were used to estimate the relative protein mobility and size.

### Pfs25 and Pfs230C Standardized ELISA

To detect vaccine-induced antibodies against Pfs25 and Pfs230C, standardized whole IgG ELISAs were performed according to a previously described protocol (17). The Pfs25 antigen was provided by Dr. Yimin Wu (NIH, USA), and the Pfs230C antigen produced using a wheat germ cell-free system (35) was provided by Prof Takafumi Tsuboi (Ehime University, Japan). For Pfs25 ELISAs, a previously reported reference serum was used (36). For Pfs230C ELISA, an internal reference serum was prepared using pooled day 70 vaccinated mouse serum with high anti-Pfs230C titers. A negative control (pooled serum from mice immunized with viral vectors expressing GFP) was included, and the optical density (OD) values for the negative controls (at 1:100 dilution) were less than 0.15 for all tested plates.

In brief, Nunc-Immuno MaxiSorp plates (Thermo Fisher Scientific, UK) were coated with monomeric Pfs25 or Pfs230C protein at 0.1 µg per well. Plates were washed and blocked. Test serum samples were diluted and added, then incubated for 2 h at room temperature (RT), and then washed again. Donkey anti-mouse total IgG conjugated to alkaline phosphatase (Jackson ImmunoResearch Laboratories, USA) was added to the plate for 1 h at RT. The plate was washed again, and a developing substrate, p-nitrophenylphosphate (Sigma-Aldrich, UK) diluted in diethanolamine buffer (Thermal Scientific, UK) was added. OD was read at 405 nm using an ELx800 absorbance microplate reader (Biotek, UK). All samples were tested against a serially diluted standard reference serum, and the OD was converted into antibody units (AUs) using a standard curve generated by the reference serum.

### Pfs28 Endpoint Titer ELISA

Endpoint titer ELISA was used to detect anti-Pfs28 antibodies, as previously described (32). In brief, Nunc-Immuno maxisorp plates were coated with Pfs28 protein (0.1 µg per well). Serum samples were added (in duplicate) and diluted threefold down the plate, followed by the same procedure as the standardized ELISA above. The endpoint titer corresponds to the *X*-axis intercept of the dilution curve at an absorbance value greater than the mean plus 3 SDs of OD for a serum sample from a naive mouse at 1:100 dilution. This method allows for comparison of anti-Pfs28 antibody titers within the study, but does not allow comparison with other studies (37, 38).

### IgG Purification

To perform SMFAs, mouse sera were pooled and the IgG purified, as previously described (39). Mouse sera from day 70 postimmunization were pooled within each test and control group. Equal volumes of serum from all mice in a group were pooled irrespective of individual antibody titer. Total IgG was purified using Protein G columns (Pierce, USA) and buffer exchanged to 1× PBS.

### Standard Membrane-Feeding Assays

Standard membrane-feeding assays measure the functional ability of vaccine-induced antibodies to block the development of *P. falciparum* strain NF54, according to a previously described standardized protocol (40, 41). Laboratory-cultured NF54 *P. falciparum* was adjusted so that the proportion of mature Stage V gametocytes was 0.15 ± 0.05%. The purified IgG was diluted into non-heat-inactivated human AB sera and mixed with the NF54 culture and fed to 4- to 6-day-old starved female *Anopheles stephensi via* a parafilm^®^ membrane. Mosquitoes were maintained at 26°C and 80% relative humidity. After 7 days, midguts from 20 mosquitoes per group were dissected to determine the number of oocysts in individual mosquitoes. Only midguts from mosquitoes with any eggs at the time of dissection (unfed mosquitoes cannot develop their eggs) were analyzed. Reduction in oocyst intensity was calculated in comparison to the respective control IgG tested in the same feed (the control was purified IgG from the group of mice vaccinated with viral vectors expressing GFP).

### Statistical Analysis

Comparison of quantitative data (e.g., day 70 antibody titers, oocyst intensities) between two groups was performed using a Mann–Whitney test and between three or more groups using a Kruskal–Wallis test. If significant, a Dunn’s multiple comparison posttest was performed.

A difference in quality of antibodies (functional activity per a fixed amount of antigen-specific antibody) judged by SMFA was evaluated using a linear regression model. The log_10_ transformed ratio of the mean oocyst count in control and test samples was the response variable, and the square root of antibody level (measured by ELISA) and IgG type (e.g., anti-Pfs25 IgG, anti-Pfs25-IMX313 IgG, anti-Pfs25-GP-Pfs230C IgG) were explanatory variables in the model. Since log of mean ratio became infinity when a test IgG had zero oocysts on average (i.e., 100% inhibition), such data were excluded from the analysis (ChAd63-MVA Pfs25-IMX313 IgG tested at 750 µg/ml of total IgG, Pfs25-GP-Pfs230C IgG tested at 375 µg/ml). All statistical tests were performed in Prism 6 (GraphPad Software Inc., USA) or JMP11 (SAS Institute Inc., USA), and *p* < 0.05 was considered significant.

## Results

### Generation and Expression of Monoantigen and Dual-Antigen Viral-Vectored Vaccines

**Figure 1:**
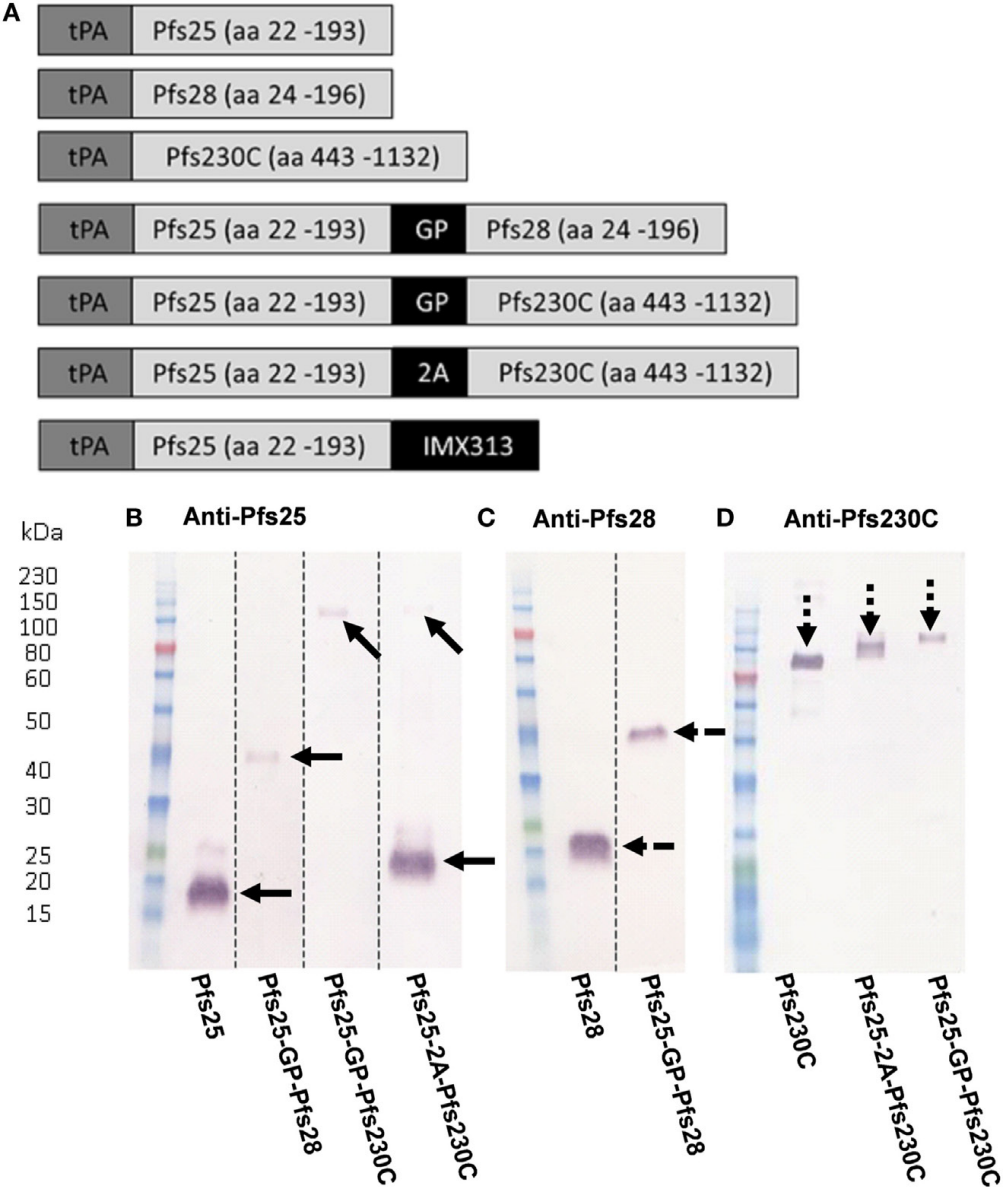
Viral vector construct generation and validation. **(A)** Seven constructs were used to generate recombinant ChAd63 and modified Vaccinia Ankara (MVA) viral vectors expressing single or multiple antigens. All antigens are fused to an *N*-terminal secretion signal peptide tissue plasminogen activator (tPA). Dual-antigen constructs link antigens with glycine–proline (GP) linker or a 2A linker. Last construct fused Pfs25 to the oligomerization domain IMX313. **(B–D)** Expression of antigens in suspension HEK293 cells. The pENTR4-LPTOS entry vector expressing the following in each lane represented: Pfs25 (18.81 kDa); Pfs25-GP-Pfs28 (38.27 kDa); Pfs25-GP-Pfs230C (98.89 kDa); Pfs25-2A-Pfs230C (19.26 kDa (Pfs25) and 83.5 kDa (Pfs230C)); Pfs28 (19.04 kDa); and Pfs230C (83.5 kDa), respectively, were used to transfect HEK293 cells, and the supernatant was harvested 4 days posttransfection. 15 µl of supernatant was loaded per lane, and after staining, the western blots were developed for 5 min. The figure shows western blots using day 70 anti-serum (pooled from five mice) collected after ChAd63-MVA vaccination against the respective antigens **(B)** Pfs25, **(C)** Pfs28, and **(D)** Pfs230C. Dotted lines indicate cut lanes from original blot **(B,C)** with the corresponding original blots shown in Figure S2 in Supplementary Material. The arrows represent the positions of the observed bands against Pfs25 (solid arrows), Pfs28 (broken arrows), and Pfs230C (dashed arrow).

A number of constructs expressing Pfs25, Pfs230C, or Pfs28 alone or in combination (shown in Figure 1A) were used to generate recombinant ChAd63 or MVA. For the constructs expressing two antigens either a G-P linker or a picornavirus 2A sequence was used between the antigens (30, 31). To test the *in vitro* expression of the antigens, pENTR4-LPTOS shuttle plasmid DNA expressing the different antigens was used to transfect HEK293 cells. The supernatant was collected and analyzed by western blot (non-reducing conditions) using polyclonal sera against Pfs25, Pfs28, and Pfs230C (Figures 1B–D). Recombinant protein for the monoantigen constructs were detected at the expected size: Pfs25 at 18.8 kDa (Figure 1B), Pfs28 at 19.0 kDa (Figure 1C), and Pfs230C at 83.5 kDa (Figure 1D).

For the fusion constructs Pfs25-GP-Pfs28 and Pfs25-GP-Pfs230C, a band was seen at the expected size of 38.27 (Figures 1B,C) and 98.89 kDa (Figures 1B,D), respectively. The Pfs25-2A-Pfs230C construct produced two cleaved proteins, Pfs25 (Figure 1B) and Pfs230C (Figure 1D). Both were detected at slightly larger sizes than expected. The observation of the higher molecular weight species in Figure 1B could be explained by the presence of the 2A sequence at the *C*-terminus as previously reported for proteins upstream of the 2A sequence (31), as this higher molecular weight species corresponds to the predicted weight for Pfs25-2A. Both Pfs25 and Pfs230C were recognized at ~100 kDa (predicted size of 105 kDa) in the Pfs25-GP-Pfs230C fusion construct (Figures 1B,D). We have previously published data on the Pfs25-IMX313 construct, which forms a heptamer of Pfs25 (16).

### Antibody Response after Administration of Monoantigen Vaccines either Mixed or Coadministered

**Figure 2:**
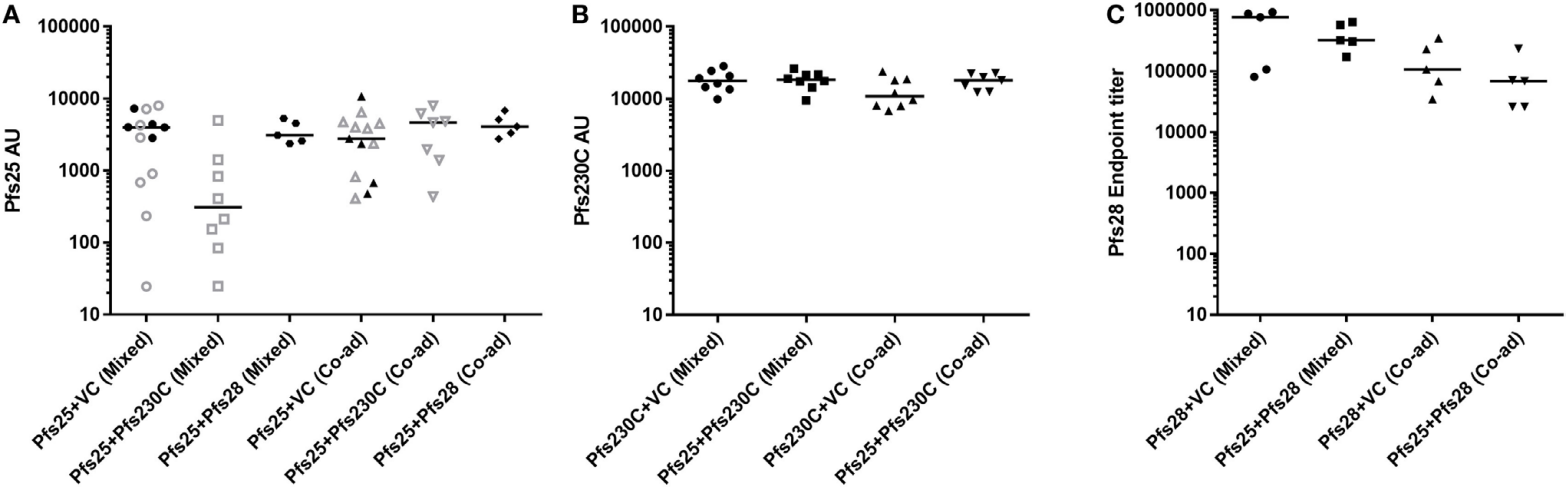
Immunogenicity of chimpanzee adenovirus 63 (ChAd63)–modified Vaccinia Ankara (MVA) vaccination with mixed or co-administered antigens in mice. Day 70 antigen-specific IgG responses in mice after immunization with ChAd63-MVA expressing Pfs25, Pfs230, Pfs28, and GFP [vector control (VC)] antigens. Constructs were administered by premixing antigens prior to vaccination (mixed) or co-administering (Co-ad) by vaccinating each antigen separately. The figure shows the total IgG response against Pfs25 [**(A)** two independent immunization experiments were conducted of experiment 1: *n* = 8/group (open symbols) and experiment 2: *n* = 5/group (closed symbols)], Pfs230C **(B)**, and Pfs28 **(C)**. Response was measured by standardized ELISA **(A,B)** and expressed in antibody units. Anti-Pfs28 antibody levels were measured by endpoint ELISA **(C)** and expressed as Pfs28 endpoint titer. Median and individual data points are shown. Kruskal–Wallis tests showed *p* = 0.0682 **(A)**, *p* = 0.2445 **(B)**, and *p* = 0.0304 **(C)**, respectively. Dunn’s multiple comparison posttests showed no significant difference.

During this study, six independent mouse immunization experiments were conducted (experiment 1–6), and the details of experiments are shown in Table S1 in Supplementary Material. To test if there was competition between the antigens, we administered the monoantigen vaccines (recombinant ChAd63 and MVA) expressing Pfs25, Pfs230C, and Pfs28 either by mixing two vaccines before administration or co-administering them at different sites. To control for the additional dose of the vaccines administered in the groups that received two antigens, we used vaccines expressing GFP as the vector control in the groups receiving vectors expressing single antigens. While Pfs25 + Pfs230C (mixed) group showed relatively lower anti-Pfs25 AU (Figure 2A), there was no significant difference among the five groups (*p* = 0.0682 by a Kruskal–Wallis test). Similarly, there was no difference in anti-Pfs230C AU (Figure 2B; *p* = 0.2445). For anti-Pfs28 endpoint titer, Kruskal–Wallis test showed a significant difference among the four groups (*p* = 0.0304), but *ad hoc* Dunn’s multiple comparison test could not identify any significant differences in any paired comparisons. Taken together, there was no obvious interference in antibody levels by receiving two antigens (mixed or co-administered) relative to groups vaccinated with vectors expressing single antigen.

### Effect of Mixing IgG against Individual Antigens on TRA

**Figure 3:**
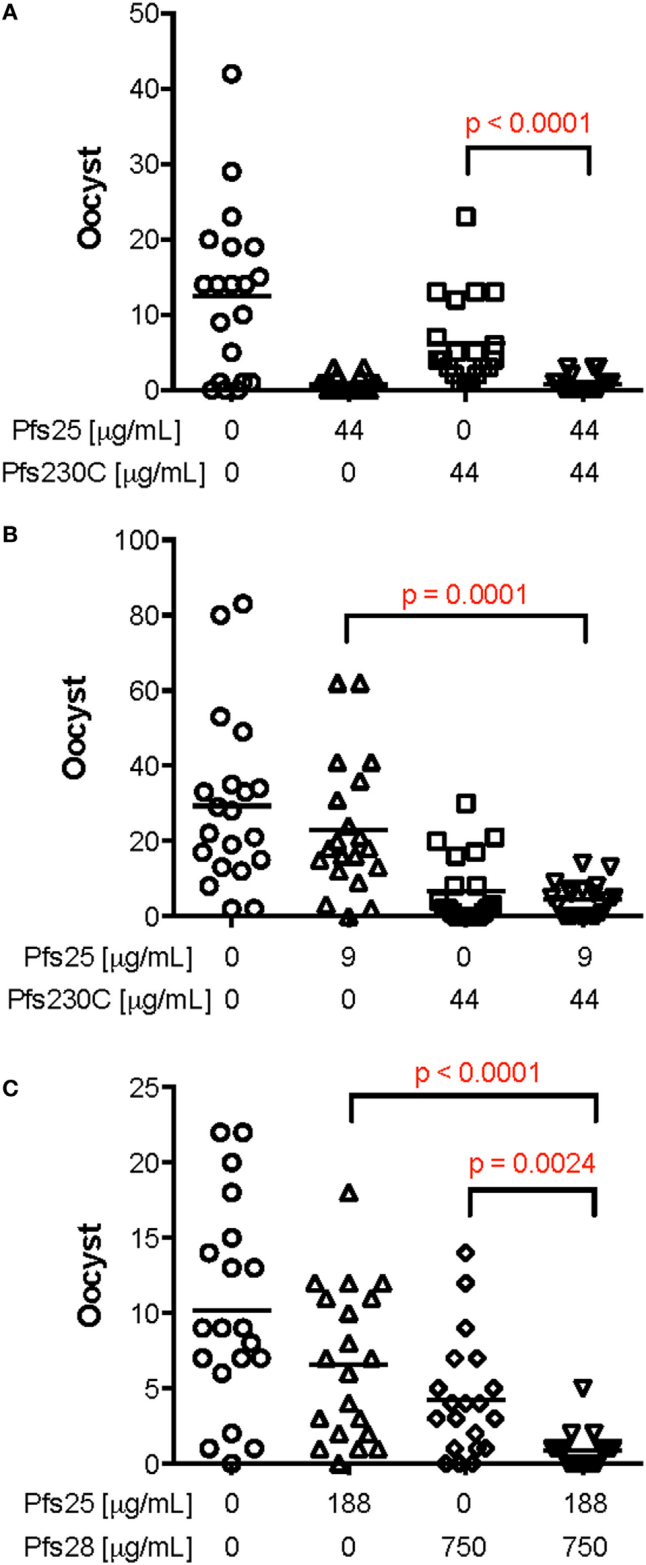
Effect of mixing mouse IgG induced by vaccination against Pfs25, Pfs230C or Pfs28 in standard membrane-feeding assay (SMFA). Total IgG was purified from pooled day 70 serum from mice vaccinated with chimpanzee adenovirus 63 (ChAd63)–modified Vaccinia Ankara (MVA) expressing Pfs25, Pfs230C, or Pfs28. The purified IgG from mice vaccinated with Pfs25 was mixed with IgG from mice vaccinated with Pfs230 [**(A,B)** the anti-sera used to purify both anti-Pfs25 and anti-Pfs230 IgGs were from experiment 3]. In addition, IgG from mice vaccinated with Pfs25 was mixed with IgG from mice vaccinated with Pfs28 [**(C)** both IgGs from experiment 4] at different concentrations. These IgG mixtures or IgG against the single antigens were fed to *Anopheles stephensi* mosquitoes in SMFA. Data points represent the number of oocysts in individual mosquitoes, and the lines show the arithmetic mean. IgG from mice immunized with ChAd63–MVA expressing green fluorescent protein were used as a negative control (the first left column in each panel). There are significant differences in oocyst density among single IgGs and IgG [*p* < 0.0001 **(A)**, *p* < 0.0001 **(B)**, and *p* < 0.0001 **(C)** by Kruskal–Wallis tests], and *p* values (Dunn’s multiple comparison posttests) are shown when there are significant differences between mixture and single IgG in each panel.

To determine whether there is interference in functional activity of the antibodies induced by monoantigen vaccines (Pfs25, Pfs230, or Pfs28), anti-Pfs25, anti-Pfs230, or anti-Pfs28 IgGs was tested either alone or in combination in SMFA (Figure 3; Table S2 in Supplementary Material). When anti-Pfs25 IgG was mixed with anti-Pfs230C IgG (44 µg/ml each), the mixture showed significantly lower oocyst intensity than anti-Pfs230C IgG alone, but there was no significant difference with anti-Pfs25 IgG alone (Figure 3A). On the other hand, when 9 µg/ml of anti-Pfs25 IgG was tested with the same concentration of anti-Pfs230 IgG, the mixture IgG showed significantly lower oocyst intensity than anti-Pfs25 IgG alone, but not to anti-Pfs230C IgG alone (Figure 3B). When a mixture of anti-Pfs25 (188 µg/ml) and anti-Pfs28 (750 µg/ml) IgGs was tested (Figure 3C), the mixture showed significantly lower oocyst intensity than either of single IgGs at that test condition. In some conditions, the mixtures of two IgG showed significantly lower oocysts than the single IgG, but not in all conditions. However, there was no significant increase in oocyst intensity (i.e., interference) by mixing two different IgGs in any conditions tested. Therefore, we next evaluated the dual-antigen vaccines for immunogenicity and functional activity.

### Immunogenicity and Functional Activity of Monoantigen and Dual-Antigen ChAd63 and MVA Vaccines

**Figure 4:**
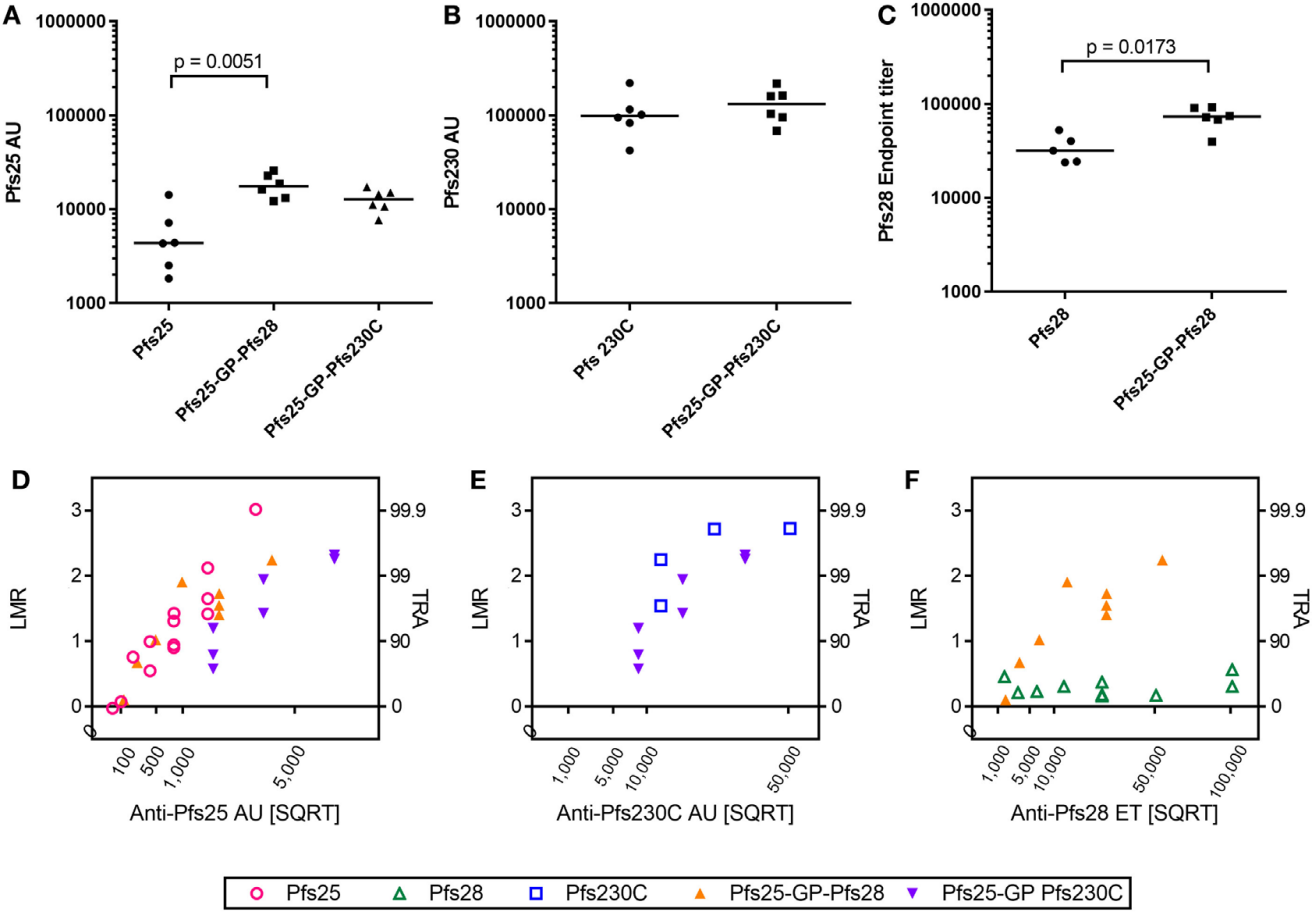
Immunogenicity and transmission reducing ability in standard membrane-feeding assay (SMFA) induced by vaccination with chimpanzee adenovirus 63 (ChAd63)–modified Vaccinia Ankara (MVA) expressing single- or dual-antigen vectors in mice. Day 70 antigen-specific IgG responses in mice after immunization with ChAd63-MVA expressing single antigens (Pfs25, Pfs230C, and Pfs28) and dual-antigen antigens linked with glycine–proline (experiment 5, *n* = 6/group). The figure shows the total IgG response against Pfs25 **(A)**, Pfs230C **(B)**, and Pfs28 **(C)**. Response was measured by standardized ELISA **(A,B)**, expressed in antibody units (AUs) and endpoint ELISA **(C)** expressed as Pfs28 endpoint titer. Median and individual data points are shown. Either a Kruskal–Wallis test followed by Dunn’s tests [**(A)** *p* = 0.0023 by a Kruskal–Wallis test, *p* values by Dunn’s tests are shown] or Mann–Whitney tests **(B,C)** were performed. Total IgG was purified from the pooled day 70 mouse serum, and each IgG was tested at three to seven different total IgG concentrations in two to five independent SMFA assays (Table S3 in Supplementary Material). The square root of antigen-specific antibody concentrations [anti-Pfs25 AU **(D)**, anti-Pfs230C AU **(E)**, or anti-Pfs28 ET **(F)**] in the feeder is shown on the *x*-axis. The log-transformed mean oocyst ratio between test and control (LMR) are plotted on left side of *y*-axis, and the associated % inhibition in oocyst density (transmission-reducing activity) is shown on the right side of the *y*-axis.

As there was no competition observed between the antigens after mixing or co-administration in terms of immunogenicity (ELISA units/titers) and functional activity (i.e., when two different IgGs were mixed in SMFA), we generated recombinant viral vectors that express two antigens from the same viral vector as described in Figure 1A. The antibody response to the individual antigens was maintained in the vectors expressing two antigens compared to monoantigen viral vectors (Pfs25, Pfs230C, and Pfs25-GP-Pfs230C in Figures 4A,B). There was a significant increase in the antibody response to Pfs25 and Pfs28 when expressed as a fusion protein compared to monoantigen vector (Figures 4A,C). This was not due to cross reactivity of antibodies in the ELISA (Figure S1 in Supplementary Material).

Since the dual-antigen vaccine groups did not show significantly lower antibodies levels compared to the monoantigen vaccine groups judged by ELISA, functionality of antibodies was compared by SMFA. Total IgG was purified from pooled day 70 serum from each group and tested at three to seven different total IgG concentrations each in two to five independent assays (Table S3 in Supplementary Material). When antigen-specific antibody concentrations were plotted in a square root scale (*x*-axis) against log-transformed mean oocyst ratio between test and control (*y*-axis), as shown in previous studies (42, 43), the data were approximated by a linear relationship (Figure 4): *r*^2^ > 0.78 for a linear fit of each IgG for each antigen, except anti-Pfs230C IgG (Figure 4E; *r*^2^ = 0.55) and anti-Pfs28 IgG (Figure 4F; *r*^2^ = 0.06).

To compare functional activity after adjusting for antigen-specific antibody levels, multiple linear regression analyses were performed using SMFA activity as a response variable, and the antibody level (anti-Pfs25AU or anti-Pfs230C AU) and IgG type (anti-Pfs25 IgG, anti-Pfs230C, anti-Pfs25-GP-Pfs230C IgG, or anti-Pfs25-GP-Pfs28 IgG) were used as explanatory variables. Since anti-Pfs28 IgG by itself showed no inhibition, a linear regression analysis was not performed for Figure 4F. The fits to the linear regression models were *r*^2^ = 0.80 and 0.81 for Figures 4D,E, respectively, and antibody levels had significant impact on SMFA activity for both analysis (*p* < 0.001 for Figures 4D,E). IgG type showed a significant impact in Figure 4D (*p* < 0.001). When the three IgG types were further compared in the linear model, anti-Pfs25-GP-Pfs230C IgG displayed significantly lower inhibitions compared to anti-Pfs25 and anti-Pfs25-GP-Pfs28 IgGs at the same anti-Pfs25 AU level (*p* < 0.001 for both). On the other hand, there was no significant impact of IgG type on inhibition in Figure 4E (*p* = 0.223).

### Comparison of Magnitude and Functional Activity of Antibodies Induced by Dual-Antigen Vaccines and Pfs25-IMX313

**Figure 5:**
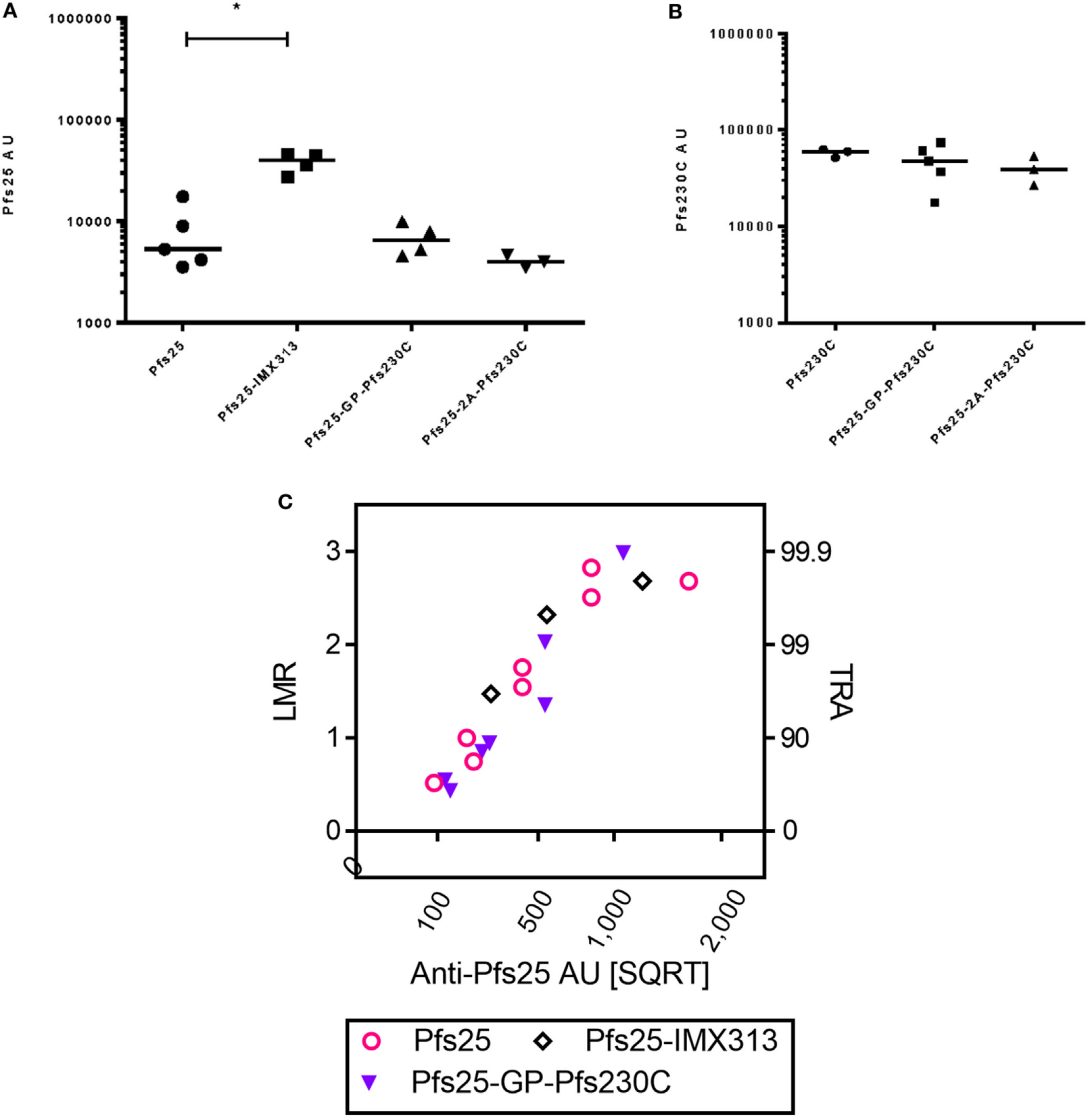
Immunogenicity and transmission reducing ability in standard membrane-feeding assay (SMFA) induced by vaccination with chimpanzee adenovirus 63 (ChAd63)–modified Vaccinia Ankara (MVA) expressing single antigen, dual-antigen, or IMX313 vectors in mice. Day 70 antigen-specific IgG responses in mice after immunization with ChAd63–MVA expressing single antigens Pfs25 or Pfs230C and dual-antigen antigens linked with glycine–proline or 2A (experiment 6, *n* = 5/group). Another group of mice were immunized with Pfs25-IMX313. The figure shows the total IgG response against Pfs25 **(A)** and Pfs230C **(B)**. Response was measured by standardized ELISA and is expressed in antibody units. Kruskal–Wallis tests [*p* = 0.0107 **(A)** and *p* = 0.4249 **(B)**] followed by Dunn’s tests were performed; *p* value by Dunn’s test is shown. Pfs25-2A-Pfs230C was eliminated from these statistical tests as only three mice remained at the end of the study. Total IgG was purified from the pooled day 70 serum, and each IgG was tested at two to five different total IgG concentrations each in two to three independent SMFA assays (Table S4 in Supplementary Material). The square root of anti-Pfs25 AU **(C)** in the feeder is shown on the *x*-axis. The log-transformed mean oocyst ratio between test and control (LMR) are plotted on left side of *y*-axis, and the associated % inhibition in oocyst density (TRA) is shown on the right side of the *y*-axis.

In addition to using the GP linker to generate dual-antigen viral vectors, we also used the picornavirus small self-cleaving 2A peptide reported to have high cleavage efficiency to express Pfs25 and Pfs230C from the same viral vectors. There was no significant difference in the antibody response against Pfs25 or Pfs230C (Figures 5A,B) when expressed using the GP linker or 2A peptide. In the same study, we also compared the immunogenicity of the dual-antigen vectors with Pfs25-IMX313, the current leading construct expressed from viral vectors (16). The anti-Pfs25 antibody response induced by vectors expressing Pfs25-IMX313 was significantly higher than Pfs25 alone, and the same trends were seen for the dual-antigen vectors.

Total IgG was purified from pooled day 70 serum from each group and tested at three to five different concentrations each in three independent assays (Table S4 in Supplementary Material). Similarly to Figure 4, the data were approximated by a linear relationship when antigen-specific antibody concentrations were plotted in a square root scale against log-transformed mean oocyst ratio between test and control (Figure 5C): *r*^2^ > 0.85 for all three groups. The overall fit to the linear regression models was *r*^2^ = 0.84, and antibody levels had significant impact on SMFA activity (*p* < 0.001), but not for IgG type (*p* = 0.691). The result indicates that when the three IgGs were tested at the same anti-Pfs25 AU level, all showed similar SMFA activity, suggesting that there is no qualitative difference in the antibodies generated by the different vaccines.

## Discussion

Transmission-blocking vaccines that combine two different antigens could have a larger impact on population transmission than a single-antigen TBV through two mechanisms: by increasing efficacy at lower antibody titers and by increasing the proportion of the vaccinated population that achieves transmission-blocking antibody levels (44). This would be further enhanced if there was synergy between the two antigens, as some preliminary evidence suggests for Pfs25 and Pfs28 (27). However, combination vaccines carry the risk of immunological interference between antigens, which could lead to reduced immune responses to one or more components (44, 45). For example, in a clinical trial that co-administered two blood-stage malaria antigens (MSP1 and AMA1), there was a significant immunological interference, with lower antibody titers of AMA1 compared to when AMA1 was administered alone (21). There is a risk of immunological interference by co-administration of two antigens (44), as reported in the blood-stage malaria vaccine clinical trial, which found a reduced immune response to AMA1 when co-administered with MSP1 (21). We showed that if two sexual-stage antigens (Pfs25 with either Pfs28 or Pfs230C) were administered to mice, either as two different vaccines given concurrently or as two different vaccines mixed and administered together, there was no significant evidence of immunological interference. We further demonstrated that two antigens co-expressed in a single viral vectored vaccine also elicited antibody-specific titers similar to a single antigen. In SMFA studies, we found that the TRA of mixing serum from mice immunized against two antigens separately was comparable to serum of mice immunized against one antigen. We found that Pfs25-GP-Pfs28 elicited Pfs25 antibody titers and Pfs28 endpoint-titers significantly higher than the respective monoantigen vaccine. However, the TRA elicited by a dual-antigen vaccine was not additive compared to one antigen, and in one case, the TRA of dual-antigen vaccine was lower than the single antigen, suggesting that dual-antigen vaccines may induce less functional antibodies. Finally, we found that the Pfs25-IMX313 vaccine elicited comparable TRA per Pfs25 AU in comparison to the dual-antigen constructs and would likely be superior due to eliciting higher Pfs25 antibody levels when compared to the dual-antigen vectors.

Antibody titers against Pfs25 and Pfs230C in this study were comparable to previous studies using a prime-boost regime with recombinant viral vectors (17). Consistent with previous studies, Pfs25-IMX313 elicited antibody titers approximately 10-fold higher than Pfs25 vaccine alone (16). This contributes to a growing body of evidence suggesting the adenovirus prime–poxvirus boost regime is an effective and versatile vaccine delivery platform for eliciting high-titer antibodies (29, 32). Viral vectors provide an ideal platform for a dual-antigen TBV, as they facilitate delivery of multiple antigens with minimal additional cost (46, 47). In our study, when comparing vaccines combining both Pfs25 and Pfs230C, there was no significant difference in antigen-specific antibody responses when they were expressed using either a GP or a picornavirus 2A sequence between the antigens (30, 31). This supports the use of both for designing future dual-antigen viral vectored vaccine constructs. Longevity of immune responses was not assessed in this study. For a practical TBV to be deployed in the field, it must induce antibody titers, which are sustained for at least one transmission season of a malaria-endemic country. Future studies will need to assess not just antibody titers, but longevity of response.

When serum from different groups of mice immunized against Pfs25 and Pfs230C individually were mixed and SMFA performed, there was no clear interference in SMFA. In some cases, mixing two antigens in certain concentrations elicited higher TRA than either antigen alone (Figure 3). However, such clear additive effects were not reproducible, and the results were difficult to generalize. Since the error of assay is not small (especially at lower % inhibition level) (42) when the assay was performed as mentioned in the current study (e.g., dissect 20 mosquitoes per group, not 100–200 mosquitoes), it is important to interpret SMFA results with caution and within context. Because of the error, it is practically difficult to determine the best combination of IgG concentrations at which we can consistently show the additive (or synergistic) effects in SMFA. Another limitation of SMFA in this study was that serum samples from multiple mice in the same group were pooled to obtain enough purified IgG for the assay. Therefore, variation among animals within a group could not be assessed.

So far, we have demonstrated that dual-antigen vaccines elicited similar antigen-specific antibody responses to monoantigen vaccines judged by ELISA (Figures 4 and 5) and that mixing antibodies against individual antigens led to at least comparable or sometimes additive TRA compared to a single antibody (Figure 3). Therefore, it was expected that IgGs from dual-antigen vaccines would show higher TRA than IgGs from monoantigen vaccines when those IgGs were tested at the same antibody level to one antigen. For example, it was expected that in the SMFA, the Pfs25-GP-Pfs230C vaccine would elicit higher TRA than the Pfs25 vaccine at the same anti-Pfs25 antibody titer, due to the additional activity from the anti-Pfs230C antibodies present. However, there was no significant increase in TRA in any of the dual-antigen vaccines compared to monoantigen vaccines when adjusted to one of the antigen-specific antibody levels (Figure 5). In fact, there was a significant decrease in TRA elicited by Pfs25-GP-Pfs230C compared to Pfs25 alone at the same Pfs25 antibody titer. There are several possible reasons for this finding. However, given that there was no significant interference when two types of IgG were mixed in *ex vivo* SMFA assays (Figure 3), it is likely that the dual-antigen vaccines induced less functional antibodies than monoantigen vaccines in that particular study experiment. On the other hand, Pfs25-GP-Pfs230C IgG showed the same level of TRA as IgG of Pfs25 alone at the same Pfs25 antibody titer from IgG samples collected from another mouse immunization study (Figure 5). Further studies are required to uncover the mechanism of non-additive effect in the dual-antigen vaccines, such as comparing TRA using affinity-purified antigen-specific IgGs from dual-antigen and monoantigen groups. It is also possible that the conformation of the individual antigens in the monoantigen and dual-antigen were different. This could result in a difference in the quality of antibodies induced, thus affecting the efficacy results observed in SMFA. We concluded this especially for Pfs25 where we observed that despite using the same concentration of anti-Pfs25 IgG in SMFA, the efficacy obtained using the dual-antigen vaccine antibodies was not higher in comparison to those of the monoantigen vaccine antibodies. However, further studies are required to determine whether there are conformational changes.

When we compared our dual-antigen vaccine with the best evidence of TRA (Pfs25-GP-Pfs230C) against Pfs25-IMX313, we found the latter induced anti-Pfs25 antibodies of the same quality but the titer was significantly higher and hence had better TRA. This suggests that despite the lack of immunological interference demonstrated with dual-antigen vaccines, they are still not as effective at blocking transmission of the parasite as vaccines, which simply elicit very high antibody titers such as Pfs25-IMX313. In some previous studies, additive/synergistic inhibition has been demonstrated when antibodies against Pfs25 and Pfs28 were combined in an SMFA (27) and when the two antigens were expressed as a fusion protein in *Saccharomyces cerevisiae* (19). In our study, we noted no strong evidence of synergy between Pfs25 and Pfs28 when expressed from the dual-antigen vaccines. This could have been as a result of the quality of antibodies raised due to the different delivery systems used in either study (viral-vectors vs. protein-in-adjuvant). There is evidence of a difference in both the quality and quantity of responses induced depending on the delivery system, viral-vectors vs. protein-in-adjuvant, with the choice of adjuvant having a role to play (48–50).

Although dual-antigen TBVs in this study did not demonstrate TRA as high as the Pfs25-IMX313 viral vectored vaccine, inclusion of multiple antigens within a TBV remains an important area of investigation. For example, future studies may consider a dual-antigen vaccine including Pfs25-IMX313 with another antigen such as Pfs230C. Furthermore, previous modeling has suggested that dual-antigen vaccines are likely to reduce the proportion of vaccinees that are poor responders (44). The significant number of poor responders to a vaccine, who fail to achieve protection, is a major potential problem for a vaccine that seeks to interrupt transmission. For example, in the 2008 Phase I trial of a Pfs25 protein-in-adjuvant vaccine, 4 of 10 volunteers developed no detectable antibodies from one dose (14). However, individuals who are poor responders to one antigen may respond better to the second antigen (provided there is no immunological interference between the antigens), which would increase the proportion of the population that achieves protection (44). Further studies are needed to determine whether expressing multiple antigens will be an effective strategy in the development of urgently needed TBVs.

Thus to address the question posed by the main the main hypothesis of this study – whether two antigens are better than one. Based on the data in this article, the answer is no. In this study viral vectors were used as the delivery platform instead of recombinant protein used in the earliest demonstration of synergy between two TBV candidates, and it is possible that repeating this study using proteins instead of viral vectors might produce a different conclusion. This study highlights the importance of assessing the effects of combining two or more antigens in a vaccine.

## Ethics Statement

All animal experiments, procedures, and handling were performed according to the UK Animals (Scientific Procedures) Act Project License (PPL 30/2414 and 30/2889) and approved by the Oxford University Local Ethical Review Committee.

## Author Contributions

VM, MK, IT, KJ, YL, and KM performed the experiments and statistical analysis. SB, FH, and CL designed the Pfs25 constructs. MK, VM and SB designed the experiments and KM, CL, and FH provided advice on study design. VM, MK, IB, KM, and SB wrote the paper. All authors reviewed the manuscript.

## Conflict of Interest Statement

FH is named on patent applications relating to vaccines and immunization regimes. FH is an employee of Imaxio SA, which owns rights to and is developing the IMX313 vaccine technology. SB is a co-founder and employee of SpyBiotech. All other authors declare that the research was conducted in the absence of any commercial or financial relationships that could be construed as a potential conflict of interest.

## References

[1] WHO. World Malaria Report 2016. Geneva, Switzerland World Health Organisation (2016).

[2] Greenwood BM. Control to elimination: implications for malaria research. Trends Parasitol (2008) 24:449–54. 10.1016/j.pt.2008.07.002 18760671

[3] Dondorp AM Yeung S White L Nguon C Day NP Socheat D Artemisinin resistance: current status and scenarios for containment. Nat Rev Microbiol (2010) 8:272–80. 10.1038/nrmicro2331 20208550

[4] WHO. Global Plan for Insecticide Resistance Management in Malaria Vectors. Geneva, Switzerland: World Health Organisation (2012).

[5] Rts SCTP. Efficacy and safety of the RTS,S/AS01 malaria vaccine during 18 months after vaccination: a phase 3 randomized, controlled trial in children and young infants at 11 African sites. PLoS Med (2014) 11:e1001685. 10.1371/journal.pmed.1001685 25072396 PMC4114488

[6] Rts SCTP. Efficacy and safety of RTS,S/AS01 malaria vaccine with or without a booster dose in infants and children in Africa: final results of a phase 3, individually randomised, controlled trial. Lancet (2015) 386:31–45. 10.1016/S0140-6736(15)60721-8 25913272 PMC5626001

[7] Moorthy VS Newman RD Okwo-Bele JM Malaria vaccine technology roadmap. Lancet (2013) 382:1700–1. 10.1016/S0140-6736(13)62238-2 24239252

[8] mal ERACGoV. A research agenda for malaria eradication: vaccines. PLoS Med (2011) 8:e1000398 10.1371/journal.pmed.1000398 21311586 PMC3026701

[9] Vogel G The ’do unto others’ malaria vaccine. Science (2010) 328:847–8. 10.1126/science.328.5980.847 20466919

[10] Kaslow DC Bathurst IC Barr PJ Malaria transmission-blocking vaccines. Trends Biotechnol (1992) 10:388–91. 10.1016/0167-7799(92)90280-9 1368880

[11] Miura K Keister D Muratova O Sattabongkot J Long C Saul A. Transmission-blocking activity induced by malaria vaccine candidates Pfs25/Pvs25 is a direct and predictable function of antibody titer. Malar J (2007) 6:107. 10.1186/1475-2875-6-107 17686163 PMC1971714

[12] Saul A. Mosquito stage, transmission blocking vaccines for malaria. Curr Opin Infect Dis (2007) 20:476–81. 10.1097/QCO.0b013e3282a95e12 17762780

[13] Nikolaeva D Draper SJ Biswas S. Toward the development of effective transmission-blocking vaccines for malaria. Expert Rev Vaccines (2015) 14:653–80. 10.1586/14760584.2015.993383 25597923

[14] Wu Y Ellis RD Shaffer D Fontes E Malkin EM Mahanty S Phase 1 trial of malaria transmission blocking vaccine candidates Pfs25 and Pvs25 formulated with montanide ISA 51. PLoS One (2008) 3:e2636. 10.1371/journal.pone.0002636 18612426 PMC2440546

[15] Ogun SA Dumon-Seignovert L Marchand JB Holder AA Hill F. The oligomerization domain of C4-binding protein (C4bp) acts as an adjuvant, and the fusion protein comprised of the 19-kilodalton merozoite surface protein 1 fused with the murine C4bp domain protects mice against malaria. Infect Immun (2008) 76:3817–23. 10.1128/IAI.01369-07 18474650 PMC2493234

[16] Li Y Leneghan DB Miura K Nikolaeva D Brian IJ Dicks MD Enhancing immunogenicity and transmission-blocking activity of malaria vaccines by fusing Pfs25 to IMX313 multimerization technology. Sci Rep (2016) 6:18848. 10.1038/srep18848 26743316 PMC4705524

[17] Kapulu MC Da DF Miura K Li Y Blagborough AM Churcher TS Comparative assessment of transmission-blocking vaccine candidates against *Plasmodium falciparum*. Sci Rep (2015) 5:11193. 10.1038/srep11193 26063320 PMC4463016

[18] Goodman AL Blagborough AM Biswas S Wu Y Hill AV Sinden RE A viral vectored prime-boost immunization regime targeting the malaria Pfs25 antigen induces transmission-blocking activity. PLoS One (2011) 6:e29428. 10.1371/journal.pone.0029428 22216279 PMC3247263

[19] Gozar MM Price VL Kaslow DC. *Saccharomyces cerevisiae*-secreted fusion proteins Pfs25 and Pfs28 elicit potent *Plasmodium falciparum* transmission-blocking antibodies in mice. Infect Immun (1998) 66:59–64. 9423839 10.1128/iai.66.1.59-64.1998 PMC107858

[20] Qian F Aebig JA Reiter K Barnafo E Zhang Y Shimp RL Jr Enhanced antibody responses to *Plasmodium falciparum* Pfs28 induced in mice by conjugation to ExoProtein A of *Pseudomonas aeruginosa* with an improved procedure. Microbes Infect (2009) 11:408–12. 10.1016/j.micinf.2008.12.009 19146977 PMC2761608

[21] Sheehy SH Duncan CJ Elias SC Choudhary P Biswas S Halstead FD ChAd63-MVA-vectored blood-stage malaria vaccines targeting MSP1 and AMA1: assessment of efficacy against mosquito bite challenge in humans. Mol Ther (2012) 20:2355–68. 10.1038/mt.2012.223 23089736 PMC3519995

[22] Forbes EK Biswas S Collins KA Gilbert SC Hill AV Draper SJ. Combining liver- and blood-stage malaria viral-vectored vaccines: investigating mechanisms of CD8+ T cell interference. J Immunol (2011) 187:3738–50. 10.4049/jimmunol.1003783 21876036 PMC3284248

[23] Chuang I Sedegah M Cicatelli S Spring M Polhemus M Tamminga C DNA prime/Adenovirus boost malaria vaccine encoding *P. falciparum* CSP and AMA1 induces sterile protection associated with cell-mediated immunity. PLoS One (2013) 8:e55571. 10.1371/journal.pone.0055571 23457473 PMC3573028

[24] Mullarkey CE Boyd A van Laarhoven A Lefevre EA Veronica Carr B Baratelli M Improved adjuvanting of seasonal influenza vaccines: preclinical studies of MVA-NP+M1 coadministration with inactivated influenza vaccine. Eur J Immunol (2013) 43:1940–52. 10.1002/eji.201242922 23589155

[25] Antrobus RD Berthoud TK Mullarkey CE Hoschler K Coughlan L Zambon M Coadministration of seasonal influenza vaccine and MVA-NP+M1 simultaneously achieves potent humoral and cell-mediated responses. Mol Ther (2014) 22:233–8. 10.1038/mt.2013.162 23831594 PMC3978791

[26] Gozar MM Muratova O Keister DB Kensil CR Price VL Kaslow DC. *Plasmodium falciparum*: immunogenicity of alum-adsorbed clinical-grade TBV25-28, a yeast-secreted malaria transmission-blocking vaccine candidate. Exp Parasitol (2001) 97:61–9. 10.1006/expr.2000.4580 11281702

[27] Duffy PE Kaslow DC. A novel malaria protein, Pfs28, and Pfs25 are genetically linked and synergistic as falciparum malaria transmission-blocking vaccines. Infect Immun (1997) 65:1109–13. 9038325 10.1128/iai.65.3.1109-1113.1997 PMC175097

[28] Sheehy SH Duncan CJ Elias SC Collins KA Ewer KJ Spencer AJ Phase Ia clinical evaluation of the *Plasmodium falciparum* blood-stage antigen MSP1 in ChAd63 and MVA vaccine vectors. Mol Ther (2011) 19:2269–76. 10.1038/mt.2011.176 21862998 PMC3242658

[29] Sheehy SH Duncan CJ Elias SC Biswas S Collins KA O’Hara GA Phase Ia clinical evaluation of the safety and immunogenicity of the *Plasmodium falciparum* blood-stage antigen AMA1 in ChAd63 and MVA vaccine vectors. PLoS One (2012) 7:e31208. 10.1371/journal.pone.0031208 22363582 PMC3283618

[30] Chia MY Hsiao SH Chan HT Do YY Huang PL Chang HW The immunogenicity of DNA constructs co-expressing GP5 and M proteins of porcine reproductive and respiratory syndrome virus conjugated by GPGP linker in pigs. Vet Microbiol (2010) 146:189–99. 10.1016/j.vetmic.2010.05.007 20570063

[31] de Felipe P Luke GA Hughes LE Gani D Halpin C Ryan MD. E unum pluribus: multiple proteins from a self-processing polyprotein. Trends Biotechnol (2006) 24:68–75. 10.1016/j.tibtech.2005.12.006 16380176

[32] Draper SJ Moore AC Goodman AL Long CA Holder AA Gilbert SC Effective induction of high-titer antibodies by viral vector vaccines. Nat Med (2008) 14:819–21. 10.1038/nm.1850 18660818 PMC4822545

[33] Cottingham MG Carroll F Morris SJ Turner AV Vaughan AM Kapulu MC Preventing spontaneous genetic rearrangements in the transgene cassettes of adenovirus vectors. Biotechnol Bioeng (2012) 109:719–28. 10.1002/bit.24342 22252512 PMC4981243

[34] Ausubel FM Brent R Kingston RE Moore DD Seidman JG Struhl K Current Protocols in Molecular Biology. New York: John Wiley & Sons (1988).

[35] Tsuboi T Takeo S Iriko H Jin L Tsuchimochi M Matsuda S Wheat germ cell-free system-based production of malaria proteins for discovery of novel vaccine candidates. Infect Immun (2008) 76:1702–8. 10.1128/IAI.01539-07 18268027 PMC2292889

[36] Wu Y Przysiecki C Flanagan E Bello-Irizarry SN Ionescu R Muratova O Sustained high-titer antibody responses induced by conjugating a malarial vaccine candidate to outer-membrane protein complex. Proc Natl Acad Sci U S A (2006) 103:18243–8. 10.1073/pnas.0608545103 17110440 PMC1636993

[37] Miura K Orcutt AC Muratova OV Miller LH Saul A Long CA. Development and characterization of a standardized ELISA including a reference serum on each plate to detect antibodies induced by experimental malaria vaccines. Vaccine (2008) 26:193–200. 10.1016/j.vaccine.2007.10.064 18054414 PMC2253722

[38] Plested JS Coull PA Gidney MAJ Elisa. Haemophilus influenzae Protocols. In: Herbert MA Hood DW Moxon ER, editors. Methods in Molecular Medicine™, vol 71 Humana Press (2003).

[39] Cheru L Wu Y Diouf A Moretz SE Muratova OV Song G The IC50 of anti-Pfs25 antibody in membrane-feeding assay varies among species. Vaccine (2010) 28:4423–9. 10.1016/j.vaccine.2010.04.036 20434549 PMC2880321

[40] van der Kolk M De Vlas SJ Saul A van de Vegte-Bolmer M Eling WM Sauerwein RW. Evaluation of the standard membrane feeding assay (SMFA) for the determination of malaria transmission-reducing activity using empirical data. Parasitology (2005) 130:13–22. 10.1017/S0031182004006067 15700753

[41] Churcher TS Blagborough AM Delves M Ramakrishnan C Kapulu MC Williams AR Measuring the blockade of malaria transmission – an analysis of the standard membrane feeding assay. Int J Parasitol (2012) 42:1037–44. 10.1016/j.ijpara.2012.09.002 23023048

[42] Miura K Deng B Tullo G Diouf A Moretz SE Locke E Qualification of standard membrane-feeding assay with *Plasmodium falciparum* malaria and potential improvements for future assays. PLoS One (2013) 8:e57909. 10.1371/journal.pone.0057909 23483940 PMC3590281

[43] Miura K Takashima E Deng B Tullo G Diouf A Moretz SE Functional comparison of *Plasmodium falciparum* transmission-blocking vaccine candidates by the standard membrane-feeding assay. Infect Immun (2013) 81:4377–82. 10.1128/IAI.01056-13 24042109 PMC3838000

[44] Saul A Fay MP. Human immunity and the design of multi-component, single target vaccines. PLoS One (2007) 2:e850. 10.1371/journal.pone.0000850 17786221 PMC1952173

[45] Sturgess AW Rush K Charbonneau RJ Lee JI West DJ Sitrin RD Haemophilus influenzae type b conjugate vaccine stability: catalytic depolymerization of PRP in the presence of aluminum hydroxide. Vaccine (1999) 17:1169–78. 10.1016/S0264-410X(98)00337-5 10195629

[46] Tine JA Lanar DE Smith DM Wellde BT Schultheiss P Ware LA NYVAC-Pf7: a poxvirus-vectored, multiantigen, multistage vaccine candidate for *Plasmodium falciparum* malaria. Infect Immun (1996) 64:3833–44. 8751936 10.1128/iai.64.9.3833-3844.1996 PMC174300

[47] Smith GL Moss B. Infectious poxvirus vectors have capacity for at least 25 000 base pairs of foreign DNA. Gene (1983) 25:21–8. 10.1016/0378-1119(83)90163-4 6229451

[48] Wang C Hart M Chui C Ajuogu A Brian IJ de Cassan SC Germinal center B cell and T follicular helper cell responses to viral vector and protein-in-adjuvant vaccines. J Immunol (2016) 197:1242–51. 10.4049/jimmunol.1502472 27412417 PMC4974488

[49] de Cassan SC Shakri AR Llewellyn D Elias SC Cho JS Goodman AL Preclinical assessment of viral vectored and protein vaccines targeting the duffy-binding protein region II of *Plasmodium Vivax*. Front Immunol (2015) 6:348. 10.3389/fimmu.2015.00348 26217340 PMC4495344

[50] Hodgson SH Choudhary P Elias SC Milne KH Rampling TW Biswas S Combining viral vectored and protein-in-adjuvant vaccines against the blood-stage malaria antigen AMA1: report on a phase 1a clinical trial. Mol Ther (2014) 22:2142–54. 10.1038/mt.2014.157 25156127 PMC4250079

