# Supplementary material for: Assessment of Antibodies Induced by Multivalent Transmission-Blocking Malaria Vaccines
Source: Front Immunol. 2018 Jan 19;8:1998. doi: 10.3389/fimmu.2017.01998 (PMC5780346; doi:10.3389/fimmu.2017.01998)
Supplement: Supplementary file 1 [file Table_1.DOCX]

**Supplementary Table 1: Outline of experiments and vaccines.**

| Experiment number | Vaccines used in experiment |
| --- | --- |
| 1 | Pfs25 ChAd63-MVA |
|  | Pfs230C ChAd63-MVA |
|  | GFP ChAd63-MVA |
| 2 | Pfs25 ChAd63-MVA |
|  | Pfs28 ChAd63-MVA |
|  | GFP ChAd63-MVA |
| 3 | Pfs25 ChAd63-MVA |
|  | Pfs230C ChAd63-MVA |
| 4 | Pfs25 ChAd63-MVA |
|  | Pfs28 ChAd63-MVA |
| 5 | Pfs25 ChAd63-MVA |
|  | Pfs28 ChAd63-MVA |
|  | Pfs230C ChAd63-MVA |
|  | Pfs25-GP-Pfs28 ChAd63-MVA |
|  | Pfs25-GP-Pfs230C ChAd63-MVA |
| 6 | Pfs25 ChAd63-MVA |
|  | Pfs25-IMX313 ChAd63-MVA |
|  | Pfs230C ChAd63-MVA |
|  | Pfs25-GP-Pfs230C ChAd63-MVA |
|  | Pfs25-2A-Pfs230C ChAd63-MVA |
