# Supplementary material for: Assessment of Antibodies Induced by Multivalent Transmission-Blocking Malaria Vaccines
Source: Front Immunol. 2018 Jan 19;8:1998. doi: 10.3389/fimmu.2017.01998 (PMC5780346; doi:10.3389/fimmu.2017.01998)
Supplement: Supplementary file 2 [file Table_2.DOCX]

**Supplementary Table 2: Oocyst inhibition in SMFA induced by mouse IgG raised against one or two mixed antigens.**

|  | Sample name | IgG conc [mg/ml] | % inhibition | | | |
| --- | --- | --- | --- | --- | --- | --- |
|  |  |  | estimate | 95%CI Lo | 95%CI Hi | p-value* |
| Feed A | Pfs230C | 0.044 | 50.00 | -43.36 | 84.03 | 0.183 |
|  | Pfs25 | 0.044 | 94.40 | 82.58 | 98.43 | 0.001 |
|  | Pfs230C + Pfs25 | 0.044+0.044 | 93.60 | 81.89 | 98.13 | 0.001 |
| Feed B | Pfs230C | 0.044 | 77.04 | 37.37 | 92.17 | 0.009 |
|  | Pfs25 | 0.009 | 21.77 | -127.27 | 72.34 | 0.671 |
|  | Pfs230C + Pfs25 | 0.044+0.009 | 85.03 | 55.00 | 94.76 | 0.001 |
| Feed C | Pfs25 | 0.188 | 58.13 | -20.95 | 86.49 | 0.115 |
|  | Pfs28 | 0.750 | 91.63 | 74.84 | 97.64 | 0.001 |
|  | Pfs25 + Pfs28 | 0.188+0.75 | 79.80 | 35.90 | 94.44 | 0.011 |
|  | Pfs25 + Pfs28 | 0.188+0.375 | 87.68 | 64.20 | 96.73 | 0.002 |
|  | Pfs25 + Pfs28 | 0.188+0.188 | 80.79 | 44.25 | 94.12 | 0.003 |

*p-value shows whether the observed inhibition was significantly (or insignificantly) different from no inhibition (control)
