# Supplementary material for: Assessment of Antibodies Induced by Multivalent Transmission-Blocking Malaria Vaccines
Source: Front Immunol. 2018 Jan 19;8:1998. doi: 10.3389/fimmu.2017.01998 (PMC5780346; doi:10.3389/fimmu.2017.01998)
Supplement: Supplementary file 3 [file Table_3.DOCX]

**Supplementary Table 3: Oocyst inhibition in SMFA induced by mouse IgG raised against single or bivalent antigens.**

|  | Sample name | IgG conc [mg/ml] | % inhibition | | | |
| --- | --- | --- | --- | --- | --- | --- |
|  |  |  | estimate | 95%CI Lo | 95%CI Hi | p-value* |
| Feed 1 | Pfs25 | 0.750 | 99.90 | 99.62 | 100.00 | 0.001 |
|  | Pfs25 | 0.375 | 96.17 | 88.94 | 98.73 | 0.001 |
|  | Pfs25 | 0.188 | 87.45 | 64.11 | 95.79 | 0.002 |
|  | Pfs230C | 0.750 | 99.95 | 99.76 | 100.00 | 0.001 |
|  | Pfs230C | 0.375 | 99.81 | 99.24 | 100.00 | 0.001 |
|  | Pfs230C | 0.188 | 97.13 | 91.21 | 99.46 | 0.001 |
|  | Pfs28 | 0.750 | 72.80 | 19.16 | 90.54 | 0.017 |
|  | Pfs28 | 0.188 | 34.58 | -93.22 | 77.00 | 0.436 |
|  | Pfs25-GP-Pfs230C | 0.750 | 99.52 | 98.39 | 100.00 | 0.001 |
|  | Pfs25-GP-Pfs230C | 0.375 | 96.26 | 89.20 | 98.78 | 0.001 |
|  | Pfs25-GP-Pfs230C | 0.188 | 73.56 | 24.13 | 90.73 | 0.011 |
|  | Pfs25-GP-Pfs28 | 0.750 | 100.00 | 99.65 | 100.00 | 0.001 |
|  | Pfs25-GP-Pfs28 | 0.375 | 99.43 | 97.65 | 100.00 | 0.001 |
|  | Pfs25-GP-Pfs28 | 0.188 | 96.07 | 87.73 | 98.91 | 0.001 |
| Feed 2 | Pfs25 | 0.750 | 100.00 | 99.54 | 100.00 | 0.001 |
|  | Pfs25 | 0.375 | 99.25 | 96.33 | 100.00 | 0.001 |
|  | Pfs25 | 0.188 | 95.09 | 86.18 | 98.46 | 0.001 |
|  | Pfs230C | 0.750 | 99.81 | 99.13 | 100.00 | 0.001 |
|  | Pfs230C | 0.375 | 100.00 | 99.51 | 100.00 | 0.001 |
|  | Pfs230C | 0.188 | 99.43 | 97.55 | 100.00 | 0.001 |
|  | Pfs28 | 0.750 | 50.94 | -51.78 | 82.06 | 0.207 |
|  | Pfs28 | 0.375 | 32.45 | -104.64 | 76.05 | 0.488 |
|  | Pfs28 | 0.188 | 31.13 | -105.63 | 77.15 | 0.474 |
|  | Pfs25-GP-Pfs230C | 0.750 | 100.00 | 99.44 | 100.00 | 0.001 |
|  | Pfs25-GP-Pfs230C | 0.375 | 98.87 | 96.73 | 99.73 | 0.001 |
|  | Pfs25-GP-Pfs230C | 0.188 | 83.96 | 51.21 | 94.58 | 0.002 |
|  | Pfs25-GP-Pfs28 | 0.750 | 100.00 | 99.39 | 100.00 | 0.001 |
|  | Pfs25-GP-Pfs28 | 0.375 | 100.00 | 99.45 | 100.00 | 0.001 |
|  | Pfs25-GP-Pfs28 | 0.188 | 97.17 | 91.36 | 99.18 | 0.001 |
| Feed 3 | Pfs25 | 0.188 | 96.27 | 88.75 | 98.82 | 0.001 |
|  | Pfs25 | 0.094 | 89.89 | 69.91 | 96.75 | 0.001 |
|  | Pfs25 | 0.047 | 82.58 | 50.31 | 94.54 | 0.002 |
|  | Pfs28 | 0.188 | 57.70 | -23.73 | 85.60 | 0.122 |
|  | Pfs28 | 0.094 | 50.86 | -42.52 | 84.22 | 0.213 |
|  | Pfs28 | 0.047 | 41.52 | -71.55 | 79.89 | 0.332 |
|  | Pfs25-GP-Pfs28 | 0.188 | 98.13 | 94.07 | 99.54 | 0.001 |
|  | Pfs25-GP-Pfs28 | 0.094 | 98.76 | 96.27 | 99.70 | 0.001 |
|  | Pfs25-GP-Pfs28 | 0.047 | 90.51 | 72.53 | 96.86 | 0.001 |
| Feed 4 | Pfs25 | 0.023 | 15.85 | -149.82 | 70.21 | 0.745 |
|  | Pfs25 | 0.012 | -6.04 | -214.63 | 60.59 | 0.868 |
|  | Pfs28 | 0.023 | 38.87 | -81.25 | 80.07 | 0.364 |
|  | Pfs28 | 0.012 | 65.28 | -1.23 | 87.92 | 0.051 |
|  | Pfs25-GP-Pfs28 | 0.023 | 78.87 | 34.29 | 93.37 | 0.002 |
|  | Pfs25-GP-Pfs28 | 0.012 | 21.51 | -137.14 | 73.17 | 0.647 |
| Feed 5 | Pfs25 | 0.375 | 97.80 | 92.70 | 99.40 | 0.001 |
|  | Pfs25 | 0.188 | 88.70 | 66.90 | 96.30 | 0.001 |
|  | Pfs25 | 0.940 | 71.70 | 19.80 | 89.90 | 0.020 |
|  | Pfs25-GP-Pfs230C | 0.750 | 99.40 | 97.20 | 100.00 | 0.001 |
|  | Pfs25-GP-Pfs230C | 0.375 | 100.00 | 99.30 | 100.00 | 0.001 |
|  | Pfs25-GP-Pfs230C | 0.188 | 93.60 | 78.70 | 98.50 | 0.001 |

*p-value shows whether the observed inhibition was significantly (or insignificantly) different from no inhibition (control)
