# Supplementary material for: Assessment of Antibodies Induced by Multivalent Transmission-Blocking Malaria Vaccines
Source: Front Immunol. 2018 Jan 19;8:1998. doi: 10.3389/fimmu.2017.01998 (PMC5780346; doi:10.3389/fimmu.2017.01998)
Supplement: Supplementary file 4 [file Table_4.DOCX]

**Supplementary Table 4: Oocyst inhibition in SMFA induced by mouse IgG raised against a single antigen, bivalent antigens or a heptamerised antigen.**

|  | Sample name | IgG conc [mg/ml] | % inhibition | | | |
| --- | --- | --- | --- | --- | --- | --- |
|  |  |  | estimate | 95%CI Lo | 95%CI Hi | p-value* |
| Feed 1 | Pfs25 | 0.750 | 99.79 | 99.28 | 100.00 | 0.001 |
|  | Pfs25 | 0.375 | 99.69 | 98.96 | 99.94 | 0.001 |
|  | Pfs25 | 0.188 | 98.25 | 94.78 | 99.46 | 0.001 |
|  | Pfs25-IMX313 | 0.750 | 100.00 | 99.59 | 100.00 | 0.001 |
|  | Pfs25-IMX313 | 0.375 | 100.00 | 99.62 | 100.00 | 0.001 |
|  | Pfs25-IMX313 | 0.188 | 99.79 | 99.21 | 100.00 | 0.001 |
|  | Pfs25-GP-Pfs230C | 0.750 | 100.00 | 99.74 | 100.00 | 0.001 |
|  | Pfs25-GP-Pfs230C | 0.375 | 99.90 | 99.50 | 100.00 | 0.001 |
|  | Pfs25-GP-Pfs230C | 0.188 | 99.07 | 96.55 | 100.00 | 0.001 |
| Feed 2 | Pfs25 | 0.083 | 90.05 | 71.69 | 96.66 | 0.001 |
|  | Pfs25-IMX313 | 0.083 | 99.53 | 97.73 | 100.00 | 0.001 |
|  | Pfs25-GP-Pfs230C | 0.083 | 86.02 | 58.63 | 95.31 | 0.001 |
| Feed 3 | Pfs25 | 0.041 | 69.80 | 12.20 | 89.79 | 0.036 |
|  | Pfs25-IMX313 | 0.041 | 96.64 | 87.69 | 100.00 | 0.001 |
|  | Pfs25-GP-Pfs230C | 0.041 | 71.81 | 17.82 | 89.89 | 0.020 |

*p-value shows whether the observed inhibition was significantly (or insignificantly) different from no inhibition (control)
