# Supplementary figures and images for: Assessment of Antibodies Induced by Multivalent Transmission-Blocking Malaria Vaccines
Source: Front Immunol. 2018 Jan 19;8:1998. doi: 10.3389/fimmu.2017.01998 (PMC5780346; doi:10.3389/fimmu.2017.01998)

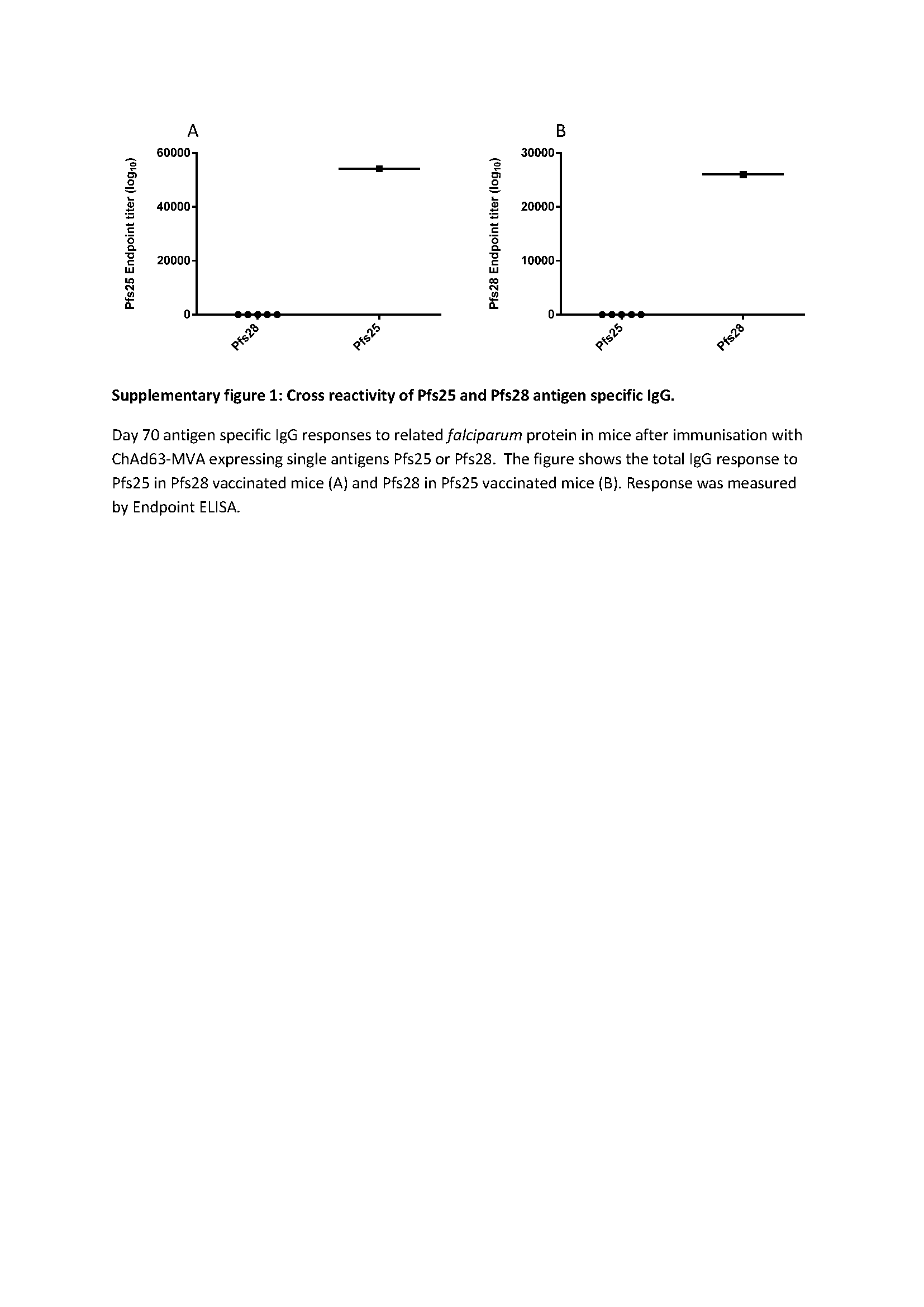

Supplement: Supplementary file 5 [file Image_1.TIF]
