# Supplementary material for: Assessment of Antibodies Induced by Multivalent Transmission-Blocking Malaria Vaccines
Source: Front Immunol. 2018 Jan 19;8:1998. doi: 10.3389/fimmu.2017.01998 (PMC5780346; doi:10.3389/fimmu.2017.01998)
Supplement: Supplementary file 6 [file Image_2.pdf]

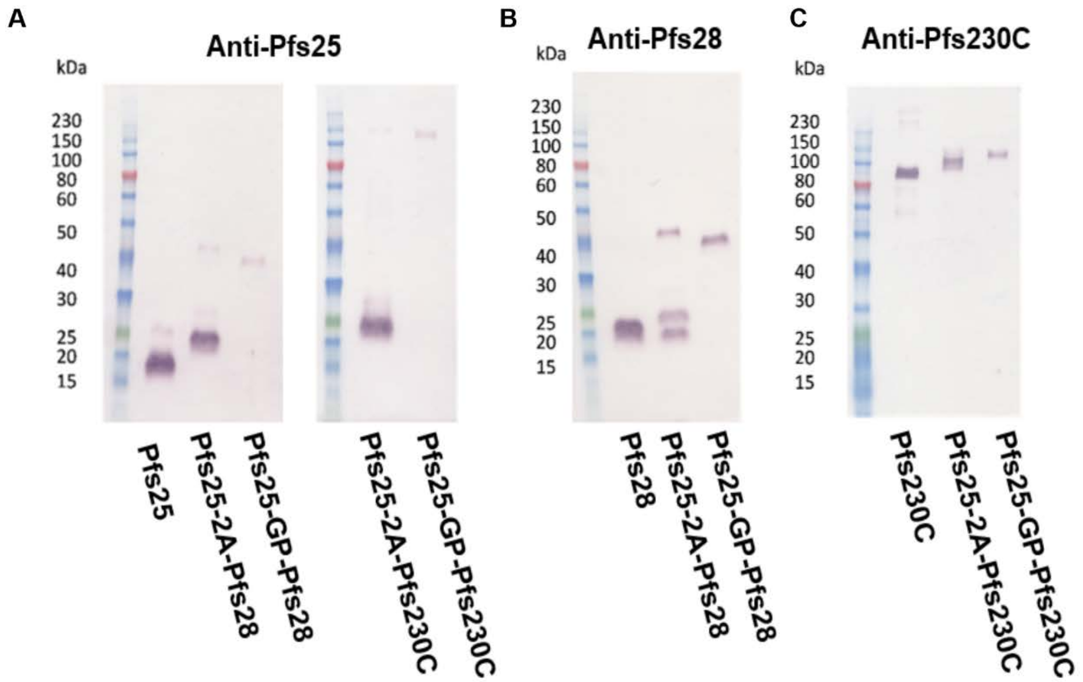

Supplementary Figure 2: Antigen expression in HEK293 cells.
Expression of antigens in suspension HEK293 cells (original western blots). The pENTR4-LPTOS entry vector expressing: Panel A – the lanes represent: Pfs25 (18.81kDa); Pfs25-2A-Pfs28 (19.26kDa (Pfs25) and 19.04kDa (Pfs28)); Pfs25-GP-Pfs28 (38.27kDa); Pfs25-GP-Pfs230C (98.89kDa); Pfs25-2A-Pfs230C (19.26kDa (Pfs25) and 83.5kDa (Pfs230C)) respectively; Panel B – the lanes represent: Pfs28 (19.04kDa); Pfs25-2A-Pfs28 (19.26kDa (Pfs25) and 19.04kDa (Pfs28)); Pfs25-GP-Pfs28 (38.27kDa) respectively; and Panel C – the lanes represent: Pfs230C (83.5kDa); Pfs25-2A-Pfs230C (19.26kDa (Pfs25) and 83.5kDa (Pfs230C)); Pfs25-GP-Pfs230C (98.89kDa) respectively. HEK293 cells were transfected with the various vectors indicated in the respective lanes and the supernatant was harvested 4 days post transfection. 15µL of supernatant was loaded per lane and after staining the western blots were developed for 5 min. The figure shows western blots using day 70 anti-serum (pooled from 5 mice) collected after ChAd63-MVA vaccination against the respective antigens (A) Pfs25, (B) Pfs28 and (C) Pfs230.
